# Disease Germs and Evolution

**Published:** 1915-05-22

**Authors:** 


### DISEASE GERMS AND EVOLUTION.

### Epidemics, War, and the Public Health.

Belief in the absolute specificity of disease was already being undermined towards the close of the last century, when the science of bacteriology was still in the early stages of development. Among those who threw doubt on the doctrine of specific diseases was one who, in 1884, wrote to the effect that if we were to believe that the germs of specific diseases were created "in the beginning, each after his kind" and had each propagated itself in strict genealogy from that time to this, then we must also believe that at the same time there were created specific susceptibilities to each of these diseases in our earliest progenitors. He maintained, on the contrary, "that starting from common ancestral conditions we arrive at specific diversities, and that the chief element in determining specificity is the nature of the soil in which the poison grows"—that is to say, on the predisposition of the individual. Although we have ceased to defend the notion of the absolute immutability of diseases, there is indeed some danger of our underestimating the relative fixity of type which some diseases have evidently acquired. This important warning was sounded by Dr. T. W. Thompson as far back as 1893.

The ever-increasing complexity of bacteriological methods has enabled us to separate organisms, apparently similar, into groups with distinct though slight differences. These differences are of varying degree, but even in the slighter cases are sufficient to satisfy the specificists. As instances may be mentioned the various groups of streptococci, the typhoid and paratyphoid organisms, and the differences distinguishing the meningococcus from the gonococcus. These detectable differences are, it must be remembered, intrinsic factors quite apart from the variations in the reactions which the same organism will produce when growing in different soils; that is, in effect, the occurrence of anomalous or aberrant cases of "specific" diseases.

It seems only common sense to apply the doctrine of evolution to diseases, for disease is but the resultant of two forces: the action of micro-organisms and the reaction of individual tissues. Both these forces are variables, and the resultants are therefore variable. It is well known that the virulence of germs can be altered by altering their environment or pabulum, and that factor which is known as the "resistance" of an individual to infection plays an even more important part in the production of what we term diseases than is often suspected.

Such considerations must be called in to explain the escape of numbers of persons exposed to scarlet fever, for instance, and the incidence of "sore throats" only in many of the contacts. It is highly probable that what we term "cerebro-spinal meningitis" is but a severe and terminal phase of an infection with the meningococcus; many cases of nasal catarrh and "sore throat" being equally results of infection with this organism. Rheumatic fever and "rheumatic tonsillitis" may also be quoted in this connection, while the relation between the gonococcus and the meningococcus may well have been an intimate one. The close resemblance between the cholera spirillum and other spirilla found in water points to the acquisition of pathogenic properties by an ordinary saprophyte.

The main theme of Haeckel's work on evolution is that in the course of their embryonic development all animals, including man, pass roughly through a series of forms which represent the succession of their ancestors in the past: "The life history of the individual is a recapitulation or epitome of the life history of the race." These are the palingenetic phenomena (*palin* = again) and those modifications due to adaptation are known as cenogenetic phenomena (*kenos* = new). It is difficult to see how either of the following alternatives can be avoided: Either pathogenic germs must have been specially created and transmitted through the ages unchanged, or they must have been evolved from a few simple ancestral forms, each succeeding generation resulting from both palingenetic and cenogenetic processes. Darwinism ascribes the main factors of evolution to the predetermination in the germ due to chance and the natural selection following on the struggle for life between individuals; the Lamarckian theory, on the other hand, gives precedence over predetermination to the influence of environment and direct adaptation of individuals to their environment. The theory of organic selection recognises evironment as a factor, and is thus a compromise, while other observers have suggested various modifications which need not be detailed here (Weismann, Spencer, etc.).

The whole question of evolution is a fascinating study, and when considered in relation to disease or to the problem of heredity in connection with disease the study becomes of immediate practical interest. Dr. J. T. C. Nash has recently published a small work* dealing with such problems, principally from the point of view of public health. The book is founded on his Chadwick Public Lectures, and traces in an unusually interesting fashion the evolutionary factors in evidence in the available records of the frightful epidemics of the Middle Ages. He also reviews some of the present day scientific evidences of evolution in relation to disease.

The difficulties surrounding the investigation of the heredity factor in the diseases of men are very great, for potential fallacies surround every inference that can be drawn. It is true that in such diseases as hæmophilia and pseudo-hypertrophic paralysis the hereditary influence seems clear, for the disease is transmitted through the mother only, and never comes from the father. Incidentally it may be remarked that these diseases seem to indicate that sex is predetermined. In the case

* *Evolution and Disease.* By J. T. C. Nash, M.D., D.P.H. Bristol: John Wright and Sons, Ltd. 1915. Price 3s. 6d.

of tuberculosis the question of the existence of hereditary influence becomes of practical importance. The more thoroughly the evidence on this score is investigated, the greater becomes the importance of the environmental factor. Darkness, dampness, and overcrowding must always be regarded as having a direct positive influence on the incidence of tuberculosis, and over and over again instances have occurred where the effect of drainage of the subsoil has been to lower tremendously the mortality from consumption.

The concluding chapter of this stimulating volume is devoted to the consideration of war as a factor in the evolution of epidemics, and the author justly observes that it will be towards the close of this exhausting war that sanitary administration will be taxed to the utmost, for the hardships of war conduce to the evolution of various forms of disease. This observation has been, as we all know, fully justified by the course of events. Care should be taken that the demands of the War Office for medical assistance are not gratified at too great a cost to the community in setting back schemes for the improvement of domestic sanitary conditions.

